# Study design to examine the potential role of assessment reactivity in the Screening, Motivational Assessment, Referral, and Treatment in Emergency Departments (SMART-ED) protocol

**DOI:** 10.1186/1940-0640-7-16

**Published:** 2012-08-28

**Authors:** Dennis M Donovan, Michael P Bogenschutz, Harold Perl, Alyssa Forcehimes, Bryon Adinoff, Raul Mandler, Neal Oden, Robrina Walker

**Affiliations:** 1 Alcohol & Drug Abuse Institute, University of Washington, 1107 NE 45th Street, Suite 120, Seattle, WA, 98105, USA; 2 Center on Alcoholism, Substance Abuse, and Addictions, University of New Mexico, 2650 Yale Street SE, Suite MSC11-6280, Albuquerque, NM, 87106, USA; 3 Center for the Clinical Trials Network, National Institute on Drug Abuse, 6001 Executive Boulevard, Room 3105, Bethesda, MD, 20892, USA; 4 Department of Psychiatry, University of Texas Southwestern Medical Center, 5323 Harry Hines Boulevard, Dallas, TX, 75390, USA; 5 The EMMES Corporation, 401 North Washington Street, #700, Rockville, MD, 20850, USA

**Keywords:** Assessment reactivity, Brief intervention, SBIRT, Clinical trials, Research design

## Abstract

**Background:**

Screening, brief intervention, and referral to treatment (SBIRT) approaches to reducing hazardous alcohol and illicit drug use have been assessed in a variety of health care settings, including primary care, trauma centers, and emergency departments. A major methodological concern in these trials, however, is “assessment reactivity,” the hypothesized impact of intensive research assessments to reduce alcohol and drug use and thus mask the purported efficacy of the interventions under scrutiny. Thus, it has been recommended that prospective research designs take assessment reactivity into account. The present article describes the design of the National Institute on Drug Abuse Clinical Trials Network protocol, Screening, Motivational Assessment, Referral, and Treatment in Emergency Departments (SMART-ED), which addresses the potential bias of assessment reactivity.

**Methods/design:**

The protocol employs a 3-arm design. Following an initial brief screening, individuals identified as positive cases are consented, asked to provide demographic and locator information, and randomly assigned to one of the three conditions: minimal screening only, screening + assessment, or screening + assessment + brief intervention. In a two-stage process, the randomization procedure first reveals whether or not the participant will be in the minimal-screening-only condition. Participants in the other two groups receive a more extensive baseline assessment before it is revealed whether they have been randomized to also receive a brief intervention. Comparing the screening only and screening + assessment conditions will allow determination of the incremental effect of assessment reactivity.

**Discussion:**

Assessment reactivity is a potential source of bias that may reduce and/or lead to an underestimation of the purported effectiveness of brief interventions. From a methodological perspective, it needs to be accounted for in research designs. The SMART-ED design offers an approach to minimize assessment reactivity as a potential source of bias. Elucidating the role of assessment reactivity may offer insights into the mechanisms underlying SBIRT as well as suggest clinical options incorporating assessment reactivity as a treatment adjunct.

**ClinicalTrials.gov Identifier:**

NCT01207791.

## Background

Since the publication of the Institute of Medicine’s report on broadening the base of treatment for alcohol problems [1], there has been a marked expansion in efforts to identify individuals with hazardous or harmful alcohol use, provide a brief intervention (BI) when indicated, and refer those with more serious levels of alcohol dependence for specialty substance abuse treatment. This process, known as screening, brief intervention, and referral to treatment (SBIRT), has been expanded more recently to also screen for illicit drugs, tobacco, and prescription medications [2,3]. It has also been implemented in a variety of health care settings where the prevalence of alcohol and drug misuse are thought to be relatively high. These have included primary care, trauma center, and emergency department (ED) settings [2,4,5].

The interventions delivered in these settings to individuals who screen positive for hazardous or harmful alcohol or drug use are often quite brief, sometimes as short as 15–20 minutes. These approaches are typically based on the principles of motivational interviewing [6-8], with feedback provided about the individual’s pattern of use and consequences derived from screening questionnaires or interviews. In clinical trials evaluating the efficacy of such interventions, extensive assessments are also conducted to elicit information characterizing the patient sample. These assessments measure substance-related and other variables that potentially moderate or mediate the effects of treatment.

Increasing concerns have been raised in SBIRT clinical trials regarding the potential impact of such assessments. The intensive assessments, relative to the brevity of the SBIRT intervention being delivered, have raised concerns that the treatment outcome may be contaminated by “assessment reactivity.” Assessment reactivity is the process by which increasing an individual’s awareness of potential problem areas by targeted and extensive assessment may, in and of itself, initiate behavior change in the absence of even a minimal intervention. Thus, it is thought that the extensive assessment may have a positive therapeutic effect and contribute to the change process [9]. While this may be beneficial from a clinical perspective, assessment reactivity may reduce the difference between the active intervention effect relative to the “inactive” control condition, thus concealing the therapeutic benefit of the active intervention [10-14]. This may have contributed to the absence of a significant difference between the intervention and “assessment only” comparison conditions in some SBIRT trials and led to an underestimation of the actual beneficial effects of BI [15].

Issues of assessment reactivity were initially noted in regard to the effects of repeated post-treatment follow-up assessments in longitudinal evaluations of alcohol treatment interventions [16-18]. A randomized study that varied both the frequency and intensity/comprehensiveness of post-treatment assessments, for example, found the poorest drinking outcomes among patients in the infrequent/low comprehensive assessment condition [17]. These findings highlighted the clinical influence of the assessment process on outcomes, and the authors also noted the lack of previous methodological designs to control or account for possible assessment reactivity.

Subsequent attention shifted from post-treatment follow-up assessment to the potential impact of assessments that take place prior to the initiation of treatment in clinical trials [19,20]. These studies found that reductions in drinking/drug use occurred between an initial screening, subsequent intake assessment, and the first therapy session. Further, these screening/post-assessment/pretreatment reductions predicted better treatment outcomes. As an example, Epstein and colleagues [19] found significant reductions in percentage of drinking days after each of the following events: a brief 10-minute telephone screening, a 90-minute clinical screening, and a 3–4 hour baseline research interview. However, there were no changes from baseline to the day before treatment or from the day before treatment to the end of the first week of treatment. Thus, the primary change in drinking behavior occurred prior to treatment initiation. While it could be argued that the observed changes reflected regression toward the mean [21], the increased attention to drinking may initiate a change process and may reflect an active therapeutic benefit of assessment [9,22]. Randomized experimental evaluations of the effects of assessment on measures of alcohol consumption among heavy-drinking college students involved in BIs have similarly found a reactivity effect [10,12,14], supporting the view that an active therapeutic process is at play rather than regression to the mean.

In contrast, studies in EDs targeting alcohol use [23,24] and marijuana [25] have not found an assessment reactivity effect. As an example, ED patients identified as having hazardous drinking patterns were randomly assigned to a screen-only, an assessment-only, or an assessment + intervention condition [23]. Drinking outcomes at a 12-month follow-up indicated that patients in both the screen-only and assessment + intervention conditions had greater, but nonsignificant, improvements compared with those in the assessment-only condition. In a study with a similar design, reductions in drinking-related outcomes were found at a 12-month follow-up among individuals identified as hazardous drinkers in the ED [24]. There were no differences, however, between patients who had been randomized to a 10–15 minute BI, a screen + assessment condition, or a screen-only group.

**Figure 1:**
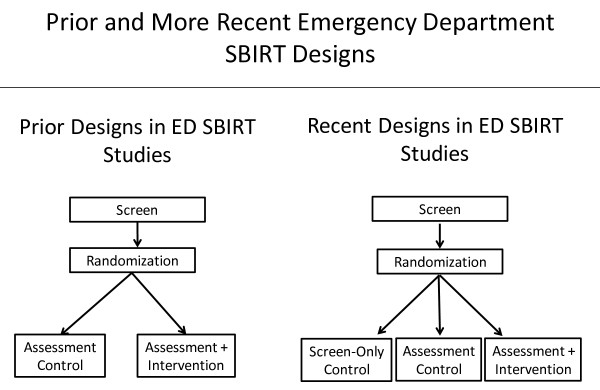
Prior and more recent emergency-department SBIRT designs.

A recent meta-analysis concluded that there have been too few high-quality, appropriately designed studies to allow any firm conclusions about the existence of assessment reactivity bias [11]. Based on these concerns, it has been recommended that researchers consider methodological approaches to identify, quantify, and minimize the potential confounding and biasing effects of assessment reactivity in any analyses targeting treatment outcomes [11,13,15,20]. Most prior studies of SBIRT, including those in EDs, have not done so. As shown in Figure 1, most prior studies have compared a screen + assessment condition with a screen + assessment + intervention condition. Taking into account the above recommendations, more recent approaches have begun to include a screen-only condition that excludes the intensive assessment component, in addition to the other two conditions [23-25]. Such a design allows one to disaggregate the impact of assessment from the combined effect of assessment plus BI. Without the inclusion of a “no assessment” or “minimal assessment” control group, it is not possible to determine how much the assessment contributed to outcome, or, in the case of a null finding, whether it masked the effects of the therapeutic intervention [11,15,26].

## Methods/design

### Overview

These concerns were taken into consideration in the design of the NIDA Clinical Trials Network’s protocol, Screening, Motivational Assessment, Referral, and Treatment in Emergency Departments (SMART-ED) [26]. The study, which is being conducted in six emergency departments across the US, is evaluating the efficacy of a motivational interviewing-based BI delivered in the ED setting, targeting individuals who screen positive for using/abusing illicit drugs or prescription medications. All study materials and procedures have been reviewed and approved by the appropriate Institutional Review Boards (IRBs) of participating universities and affiliated EDs. The trial will be conducted in compliance with protocol, International Conference on Harmonisation Good Clinical Practice guidelines, and applicable regulatory requirements. An independent Data and Safety Monitoring Board oversees the study.

**Figure 2:**
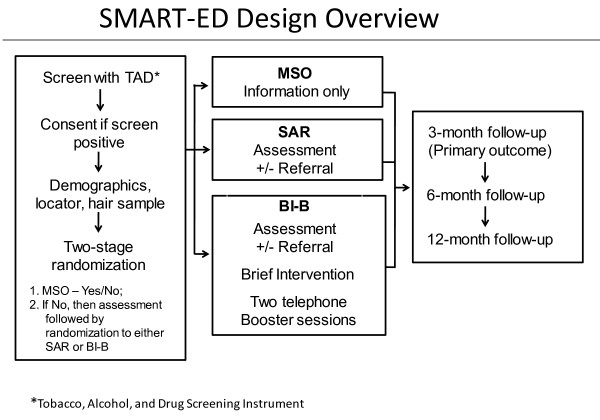
SMART-ED design overview.

In order to assess the independent contributions of assessment and the BI to outcomes in the study population, a three-arm design was adopted. The three treatment groups are (1) a minimal screen-only condition (MSO) in which participants receive as little interaction as possible and do not complete an intensive baseline assessment; (2) screening, assessment, and (if indicated or requested) referral to treatment (SAR); and (3) screening, assessment, and (if indicated or requested) referral, plus BI lasting up to 30 minutes in the ED followed by two telephone booster intervention sessions within 1–4 weeks following the ED visit (BI-B). Figure 2 presents an overview of this design.

### Primary hypotheses

There are three primary hypotheses: (1) Participants randomized to BI-B will have fewer days of use of the patient-defined primary problem substance during the 30 days prior to the 3-month assessment relative to participants randomized to MSO; (2) participants randomized to BI-B will have fewer days of use relative to participants randomized to SAR; and (3) participants randomized to SAR will have fewer days of use relative to participants randomized to MSO.

The study also has a number of secondary objectives: (1) To evaluate the effect of BI-B relative to SAR on substance abuse treatment engagement among those participants who are referred to treatment; (2) to evaluate the effect of the experimental interventions on self-reported health-care utilization; (3) to evaluate several putative predictors of outcome (patient demographics, substance use severity at baseline, primary substance of abuse, motivation, therapeutic alliance, perceived substance-relatedness of ED visit, and psychiatric comorbidity) in terms of both main effect and interaction with treatment assignment; and (4) among participants assigned to BI-B, to evaluate dose–response to the number of booster sessions received.

### Study population: Inclusion/exclusion criteria

To be included in the trial, individuals need to meet the following criteria: (1) registration as patient in the ED during study screening hours; (2) positive screen (≥3) on the 10-item Drug Abuse Screening Test (DAST-10) for problematic use of a nonalcohol, non-nicotine drug; (3) at least one day of problematic drug use (excluding alcohol and nicotine) in the past 30 days; (4) age 18 years or older; (5) adequate English proficiency and literacy; (6) ability to provide informed consent; and (7) access to phone (for booster sessions). Individuals are excluded from consideration if they meet any of the following criteria: (1) Inability to participate due to emergency treatment; (2) significant impairment of cognition or judgment rendering the person incapable of informed consent (e.g., traumatic brain injury, delirium, intoxication); (3) status as a prisoner or in police custody at the time of treatment; (4) current engagement in addiction treatment; (5) residence more than 50 miles from the location of follow-up visits; (6) inability to provide sufficient contact information (e.g., at least 2 reliable locators); and (7) prior participation in the current study.

Emergency department patients who are subsequently hospitalized may differ from other ED patients in ways that could affect outcome and response to treatment. For example, they would likely have more severe medical conditions, which could affect motivation to change, and may have greater exposure to treatment and referral while in the hospital. Since there is no reliable way to know which patients will be hospitalized at the time of randomization, hospitalized patients will be kept in the study. The ED logs will be reviewed to identify all participants who are ultimately hospitalized as a result of their ED visit. Secondary analyses will explore whether such patients differ from nonhospitalized participants in substance use outcome or treatment response.

### Characteristics of participating emergency departments

To be included as potential sites for this trial, EDs should have a large volume of patients who use drugs and that represent the US population in aggregate, have prior research experience, and not currently be routinely conducting SBIRT targeting drug users. Further, they should be able to present a convincing plan for patient flow and use of space, have (or are able to hire) appropriate staff to conduct the study, have a sufficient referral network for patients needing specialty addiction treatment, and have an ED physician who can serve as protocol PI (or otherwise be actively involved in the protocol). From a pool of 17 EDs that responded to an initial site-survey request, and following review of the above criteria and subsequent site visits to a smaller subset of potential sites, six EDs were selected to participate in the trial.

### Primary and secondary outcome measures

The primary outcome for the study is the number of days of use of the patient-defined primary problem drug, assessed for the 30-day period preceding the 3-month post-intervention follow-up, as assessed by the Timeline Followback (TLFB) [27,28].

In addition, a number of secondary outcomes are included: (1) number of days of primary problem substance use during the 30 days preceding the 6- and 12-month postintervention follow-ups; (2) days of abstinence from all drug use during the 30 days preceding the 3-, 6-, and 12-month postintervention follow-ups; (3) days of heavy drinking in the past 30 days at 3, 6, and 12 months; (4) quantity of use (dollar value, number of times used) for the primary drug at 3, 6, and 12 months from baseline; (5) change in days of use of the primary drug between baseline and follow-up at 3, 6, and 12 months (only for the SAR and BI-B groups that completed the TLFB at baseline); (5) relative change in hair-sample drug screen results for the primary drug and also for any drug of abuse from baseline to 3, 6, and 12 months postintervention; (6) consequences of drug use (based on the NIDA-Modified Alcohol, Smoking and Substance Involvement Screening Test (NM-ASSIST) [29]; (7) participation in addiction treatment in the past 30 days at 3, 6, and 12 months postintervention among SAR and BI-B participants with probable dependence who are referred for treatment (assessed with the Treatment Services Review [TSR]) [30]; and (8) health-care utilization (number of ED visits, number of hospital days, and number of outpatient visits) in the past 30 days at 3, 6, and 12 months based on TSR results.

### Screening of potential participants

During defined recruitment hours, Research Assistants (RAs) will identify possibly eligible patients seen in the ED and will approach them about their willingness to participate in anonymous screening to determine the representativeness of the study sample. Participants will provide verbal (not signed) consent using a brief IRB-approved script for the anonymous collection of screening data, which includes age, gender, and reason for ED visit. The RA will attempt to screen all ED patients until recruitment hours have ended or the maximum recruitment for the week is achieved (no more than 12 participants randomized per week per site). Refusals and inability to participate (e.g., unavailable due to emergency medical treatment, left without being seen) will be recorded.

Study staff will time their involvement with patients to minimize interference with medical treatment. Depending on level of acuity, some patients will be approached prior to the initial evaluation by a physician, and some after. Patients who show signs of intoxication (e.g., somnolence, slurring of speech) will not be screened unless these signs resolve. Screening data will be collected by research personnel and by participant self-report using direct entry into tablet computers to facilitate rapid screening, electronic data capture, and mobility within the busy ED setting.

The logistics of conducting screening in the ED require that the screening process be relatively brief and minimally intrusive. Further, to minimize potential assessment reactivity in the MSO group, substance-related screening questions were to be kept at an absolute minimum. A four-section screening instrument, the Tobacco, Alcohol, and Drug (TAD) questionnaire, will be used to capture the substances assessed. To maximize confidentiality, the TAD will be completed by the participants, who will enter the data directly using the tablet PC unless the participant is not comfortable with this technology, in which case the data may be entered by the RA. The four sections, which can be completed in 5–10 minutes, consist of (1) the 4-item Heavy Smoking Index [31], a brief screen for nicotine dependence that is administered first to minimize participant discomfort with answering more sensitive questions about drug use; (2) the three consumption items of the Alcohol Use Disorders Identification Test (AUDIT-C) [32,33]; (3) the DAST-10 [34-36]; and (4), for individuals scoring ≥3 on the DAST-10, three questions to determine primary substance of abuse, days of use of the primary substance, and substance-relatedness of the ED visit. The screen is considered positive if the DAST-10 score is ≥ 3 *and* the individual reports past 30-day use of the primary substance.

Participants who meet screening criteria based on the TAD will be further screened for eligibility by the RA, who will ask participants about (1) current engagement in addiction treatment, (2) whether they reside more than 50 miles from the follow-up location, (3) ability to provide at least two locators, and (4) access to a phone. These two prescreening forms (the TAD and eligibility) will not include identifying information. Individuals who are interested and eligible to participate will then undergo the informed consent process. Patients who do not meet inclusion criteria will receive no further intervention, but the anonymous screening data will be kept to allow comparison to those who do meet criteria.

### Informed consent process

Patients will be provided with an IRB-approved informed-consent form that will include a description of all significant elements of the study. The consent form will also include assurances of confidentiality and a statement that participation is entirely voluntary, that the decision to participate will in no way influence other aspects of the patient’s treatment, and that the participant is free to withdraw participation at any time. Patients must be able to read the first paragraph of the informed consent and express verbally their understanding of the key elements of the study (e.g., random assignment, possible interventions received, duration of follow-up). They will then indicate their consent to participate in the study by signing and returning the informed consent form. A Health Insurance Portability and Accountability Act (HIPAA) disclosure form will also be required to allow the study to access protected health information in the patient’s medical record in the ED. Because of the time constraints of completing the assessment and intervention in the ED, and because of the relatively low risks associated with the study, the consent form will be as brief as possible within the constraints of adequate human subject protections. Prior to randomization, all consenting participants will complete a demographic questionnaire, provide locator information, and provide a hair sample, which will be used to obtain an objective measure of substance use.

### Randomization procedure

After providing informed consent, all eligible participants will be randomly assigned in 1:1:1 ratio to one of the three conditions (MSO, SAR, or BI-B). Randomization will be stratified based on site, presence of an alcohol use disorder (AUDIT-C score ≥ 4), and drug problem severity (DAST-10 score ≥ 8). The randomization procedure will be conducted through a centralized, web-based process set up by the NIDA Clinical Trials Network Data and Statistics Center. The randomization sequence will be unknown to staff, and treatment assignment will be revealed in two stages. Initially, the RA performing the randomization will be informed as to whether the participant is in the MSO group or not. If so, the RA is finished with the research component in the ED and will have no further study involvement until the 3-month follow-up visit. The remaining participants will receive a more extensive baseline assessment. After completion of the baseline assessment, RAs will be informed whether participants are in the SAR or BI-B group. Research assistants conducting the follow-up assessments will be blinded to treatment condition. The randomization scheme was designed to balance patient allocation to each treatment arm. Given the intent-to-treat nature of the study, participant dropouts after randomization will not be replaced.

### Baseline assessments (SAR and BI-B groups only)

Given both the concerns about the potential effects of assessment reactivity and the practical difficulties associated with an extensive assessment procedure (e.g., interfering with the rapid pace of clinical treatment in the ED setting), the baseline assessment is shorter than that often found in addiction treatment trials. The semistructured 30-day TLFB interview [27,28] is the measure from which the primary outcome will be derived. The TLFB, which uses a calendar prompt and a number of other memory aids to facilitate accurate recall, provides estimates of the daily quantity, frequency, and pattern of substance use (cannabis, cocaine, methamphetamine and prescription stimulants, heroin and prescription opioids, inhalants, sedatives, hallucinogens, alcohol, tobacco, and other drugs) during a specified time period. For each day of use, the RA also will record both the number of times the substance was used and the dollar value of the amount of substance consumed. The baseline assessment also will include the NM-ASSIST [29,37] to assess frequency of use and associated problems for a number of substances including tobacco, alcohol, cannabis, cocaine, amphetamine-type stimulants, inhalants, sedatives, hallucinogens, opioids, and other drugs. The NM-ASSIST includes a separate assessment of prescription stimulants and methamphetamine and of prescription opioids and heroin. The total time burden for the baseline assessment is approximately 30 minutes.

Hair testing will be used at baseline (following consent and prior to assessments) and at 3-, 6-, and 12-month follow-ups as an objective secondary measure of cannabis, cocaine, amphetamine, methamphetamine, opioid, and benzodiazepine use. Compared with body fluids such as urine or saliva, hair testing enables the detection of substance use over a significantly longer window of time [38,39] and is increasingly being used as an objective approach complementary to self-reported substance use outcomes in clinical trials. A significant benefit of this testing approach is the comparatively nonintrusive nature of collecting a hair sample from the scalp [40].

### Study interventions

#### Minimal screening only (MSO)

Following randomization, participants in the MSO group will receive an informational pamphlet about drug use and its consequences, addiction, and treatment. This pamphlet will consist of information collected from materials produced by NIDA for the general public. They will receive no further intervention.

#### Screening and referral (SAR)

Participants in the SAR group will receive the same informational pamphlet as the MSO group. Those with probable dependence (NM-ASSIST score ≥27 for any drug or alcohol) will also be provided with minimal scripted feedback to let them know their score is in the high-risk range, and will receive a recommendation to seek treatment. The RA will provide these participants with an information sheet listing treatment and self-help resources in their community. The latter will be standardized in format but will include site-specific information, i.e., names, addresses, and phone numbers of local addiction treatment agencies in the normal clinical referral network of the participating EDs. Based on our initial site survey, the majority of responding EDs are not currently screening or referring systematically, so the SAR condition represents a level of care significantly higher than treatment-as-usual.

#### Brief Intervention + booster (BI-B)

Individuals randomized to the BI-B condition will receive the same information and referral as those in SAR. However, in addition to these materials, while in the ED the BI-B group will receive a manual-guided BI based on motivational interviewing (MI) principles, including feedback based on screening information, the Feedback, Responsibility, Advice, Menu of options, Empathy, Self-efficacy (FRAMES) heuristic, and development of a change plan, as delivered in previous trauma center trials [6,41-44] and the Washington State SBIRT projects [6,41,42,45-49]. As Sise [50] indicated, SBIRT services such as these can be integrated effectively into all components of a busy urban ED by adding specially trained interventionists to the ED service staff and/or by training existing staff. The BI will be performed by study RAs (rather than regular ED staff members) who have received centralized training, have successfully completed and met criterion skill levels as assessed by objective ratings of tapes of their interventions with a series of subsequent training cases, and who will receive ongoing fidelity monitoring and clinical supervision over the course of the trial. For a given study participant, BI will be performed by a different RA than the one who conducted assessments of that individual. The in-person BI, which will be approximately 30 minutes long, will be delivered while the participant is still in the ED. Participants in BI-B will receive the informational pamphlet and referral for treatment (if indicated) from the BI interventionist.

In addition, as a means of augmenting the intervention in the ED, participants also will receive up to 2 phone “booster” sessions. These sessions, which are also based on MI principles, will check to see whether they have engaged in treatment, review change plans, and seek a commitment from them [51]. This number of booster sessions was chosen to replicate the structure of the standard Motivational Enhancement Therapy (MET) [52], with the goal of maximizing the magnitude of the therapeutic effect while keeping the intervention short enough to be practical. The goal of the first phone session is to re-engage and reinforce the change plan and support continuing change efforts. The second phone session is a check-in and addresses barriers to change or treatment engagement. Each of these booster calls will be approximately 20 minutes long. The content of these boosters is patterned after sessions in MET [52,53]. The phone booster process is similar to those previously used to deal with problem drinkers in EDs, either as part of stepped-care interventions initiated in the ED and continued following discharge [54] or as stand-alone phone-delivered BIs conducted without an initial intervention in the ED [51], and to procedures used in a primary care settings [55]. The target window for the initial first and second phone booster calls is within 3 days and within 7 days of discharge from the ED, respectively. If initial attempts to complete the booster sessions within these windows are unsuccessful, further attempts to engage participants will be made for up to one month post-discharge from the ED. Booster calls will be made from a centralized, study-wide intervention booster call center by interventionists who have received standardized training and will have ongoing fidelity monitoring and clinical supervision.

### Follow-up assessments

Follow-up assessments will be conducted at 3, 6, and 12 months following the ED visit. In order to maintain blinded follow-up, different RAs will be used to complete baseline and follow-up assessments. If in-person follow-up is not possible, follow-up visits may be conducted by phone, but this should be avoided if possible, as hair samples would be missing in such cases. In addition to measures of substance use (e.g., NM-ASSIST, TLFB, hair sample), the Treatment Services Review (TSR) [30] also will be administered at these follow-up points to track substance-abuse treatment entry and utilization and medical-treatment service utilization.

Follow-up will be conducted in person at a central location, preferably close to the participating ED. Additionally, if participants are unable to provide their own transportation to or from follow-up visits, sites may provide transportation including bus or taxi fare if they have local IRB approval to do so. As a means to achieve high follow-up rates in the study population, adequate compensation for time and inconvenience is critical. Compensation of $50 for the screening/baseline visit and $75 for the 3-month visit (primary outcome) and subsequent visits is proposed.

### Safety assessment

Adverse events, serious adverse events, and substance use events (e.g., worsening of drug use, need for higher level of care, admission to detox or inpatient drug treatment) will be monitored and reported throughout the study. These events will be subject to ongoing monitoring by the study executive committee and will be presented for review by the independent Data and Safety Monitoring Board.

### Blinding

As in almost all psychosocial treatment protocols, study participants will not be blinded. However, because of the two-stage method of revealing the randomized group assignments, RAs conducting baseline assessments will be partially blinded until the assessment has been completed. The RAs conducting follow-up assessments will be different from those conducting the baseline assessments and will be blinded to treatment condition. To further minimize potential bias, the RA who conducts the baseline assessment in the ED will not be the same one that conducts the BI-B intervention with that patient.

### Sample size determination

There is no straightforward way to estimate the effect size of the proposed contrasts across the three conditions due to differences across critical aspects of prior SBIRT studies (e.g., primary care setting rather than ED, or alcohol versus drug use as the primary outcome). While previously published studies guided our estimates of treatment effect, the power analysis and sample size calculations were based on the minimum clinically significant nonstandardized effect that we wish to be able to detect between SAR and BI-B, and estimates of error variance based on the data collected in the Washington State implementation of an SBIRT model in ED setting [48,56]. In order to detect an absolute difference of three days between the two groups, which was considered to be a clinically meaningful difference, and with an estimated standard deviation of 11 days, an anticipated 15% attrition rate, and a Type I error *α* = .05/3 (two-tailed), a total of 1285 subjects (429 in each arm) was determined necessary to have 90% power. Estimating conservatively that 5% of screened patients will meet inclusion criteria, and that 75% of these will consent to participate in the study, it will be necessary to screen 34,267 patients (5711 per site) to achieve the proposed sample of 1285 randomized participants. We propose to screen approximately 160 individuals per week per site to achieve the targeted enrollment rate of six participants per week per site. This will allow recruitment at each site to be accomplished within nine months.

### Statistical methods for primary analyses

The primary analyses will consist of three prespecified pair-wise comparisons between the MSO, SAR, and BI-B conditions with respect to the primary outcome variable (number of days of use of the primary drug of abuse in the past 30 days at three-month follow-up based on the TLFB), all of which will use a simple closed testing procedure to control family-wide Type I error at no more than 0.05. Linear mixed-model analyses with a random site effect and fixed treatment effect and intercept will be used. These analyses will take into account possible variability in the overall level of abstinence between the sites, possible site-by-treatment interaction, and the level of a baseline covariate (days of use of the primary drug of abuse, as defined at screening by the DAST-10, during the 30 days preceding the baseline assessment). The primary outcome will be analyzed according to the intent-to-treat principle. Patients who refuse treatment will still be followed for outcome in their assigned group, but no attempt will be made to impute outcomes for these patients.

### Potential interpretations of prespecified group comparisons

The proposed design provides the opportunity to make a number of meaningful comparisons. First, the contrast between the SAR and BI-B groups allows a determination of the incremental benefit of the assessment + BI + booster sessions over and above the effects of the assessment alone. This contrast has been conducted in most prior SBIRT studies and is the one for which the group differences and effect size may be attenuated due to potential assessment reactivity. The second comparison, between the SAR and MSO, allows the determination of any incremental therapeutic effects of assessment over and above minimal contact. This comparison provides the quantification of the assessment reactivity effect. The final comparison, between the BI-B and MSO groups, provides a measure of the total effect of the intervention, including any therapeutic effects of assessment and referral. The MSO condition actually offers a better approximation of true treatment-as-usual, as opposed to SAR, since the latter condition represents a greater intensity of intervention than is typically found in EDs.

There are many potential equalities and inequalities that can hold between the three arms of the SMART-ED design. Assuming that abstinence is the outcome (and the bigger the better), the most intuitive results of possible comparisons and appropriate conclusions are depicted in Table 1. For example, if MSO < SAR < BI-B, we may conclude that there is both assessment reactivity and a treatment effect. Other rows can be interpreted similarly. Thus, each significant difference, or lack of difference, between groups would provide meaningful evidence of the presence or absence of assessment and/or treatment effects but with different implications for practice.

**Table 1:** Interpretation of potential assessment and treatment effects based on outcomes of contrasts among SMART-ED conditions

**Equalities and Inequalities**					**Conclusions**	
MSO^*^		SAR**		SAR/BI-B†	Assessment Reactivity	Treatment Effects
	<		<		Yes	Yes
	<		=		Yes	No
	=		<		No	Yes
	=		=		No	No

*MSO = minimal screen only. **SAR = screening, assessment, and referral to treatment if indicated or requested. †SAR/BI-B = screening, assessment, and referral if indicated or requested plus brief intervention and subsequent telephone follow-up booster sessions.

While assessment has typically been an integral component of BI, in a number of general health-care settings it is likely that BI may be conducted without assessment; there is often limited or no time for conducting an assessment beyond a brief screen in such settings, and assessment is often seen as complex by generalist clinicians. The possibility and effectiveness of conducting BIs without assessment is a condition that is not examined in the current design. Conversely, it is possible to have assessment without treatment. If the SAR condition has a better outcome than the minimal screen only (MSO) condition, and the BI-B condition does not outperform SAR, then a case could be made for simply conducting an assessment and subsequent referral to specialty substance abuse treatment. Given the added costs involved, an intervention should be avoided if it does not significantly add to the outcome over and above that of SAR.

## Discussion

While the three-arm design follows the recommendation for taking potential assessment reactivity into account, there are a number of limitations that need to be noted. One obvious limitation is that the effects of the minimal screening, which may not be negligible, cannot be assessed [26]. A number of screening instruments that have been developed for use in SBIRT trials consist of as few as one item [57,58]. Although the MSO condition attempts to minimize the length of the screening procedure and measure its impact, it nevertheless consists of 20 items, including the 4-item Heavy Smoking Index [59], the AUDIT-C [32], the DAST-10 [34], and the 3 questions on primary substance of abuse, days of use, and substance-relatedness of the ED visit. A recent meta-analysis and systematic review of BI [13] showed that merely asking questions about alcohol use alters subsequent drinking behavior even in the absence of a more intensive assessment or intervention.

Second, another approach to reducing bias in SBIRT trials may be to blind subjects to study purpose as a way of limiting the potential effects of social desirability [15]. The SMART-ED design does not address this issue specifically; however, participants and staff are blinded to final condition assignment via the two-step process of revealing the randomization results. The RA who conducts the screening and baseline assessment and who is made aware of the randomized treatment assignment “hands off” the patient to a different RA who delivers the assigned intervention, thus minimizing the likelihood that participants will be treated differently (e.g. subtly advising MSO subjects to change). Further, research staff who conduct follow-up assessments are different from those who conduct baseline assessments or interventions in the ED and are blind to participants’ treatment condition.

Blinding subjects to study purpose is difficult given both the need for informed consent and the nature and content of the screening instrument. It is not possible to determine what impact the consent process has on outcomes given that, in the current trial (as in many similar trials), the IRBs require disclosure of the purpose and procedures of the study. The present study was granted IRB approval to use an abbreviated script to approach potential participants in the ED and ask if they would be willing to answer the TAD screening question; only verbal (not signed) consent was required. Even this procedure, however, alerts individuals to the intended focus on substance use. The collection of hair samples following completion of the screening questionnaire by all potential subjects but prior to obtaining formal written consent may alter individuals’ willingness to volunteer for the study. Similarly, the collection of hair samples following the screen but prior to the baseline assessment for the SAR and BI-B groups might alter responses across these two sets of drug-use data.

While research ethics need to be followed, methods to increase the blinding of potential subjects in BI studies have been approved by IRBs. As an example, patients in a Level-I trauma center who screened positive for hazardous or harmful alcohol use were asked to give consent to assess their long-term outcome from trauma [44]. Assessments included 6- and 12-month follow-up interviews and review of their medical records and other databases. Consent was not obtained for randomization into an SBIRT intervention or control group, nor were patients told they were taking part in a study to reduce alcohol consumption. After consent, patients were randomized to an intervention or control group. Such a procedure avoided the bias that otherwise might have occurred because more seriously alcohol-impaired or dependent patients may have refused to participate, and patients who did participate would have been sensitized to the fact that their drinking would be monitored [15]. Finally, it is unclear to what extent the presence or absence of assessment reactivity in SMART-ED can be generalized to other trials with other assessments.

Although there are questions about whether assessment reactivity effects occur in BI studies in general [11], and, more specifically, in those delivered in EDs [23-25], it is important to use designs that take this possibility into account. As noted, a number of methodological factors may contribute to biases that can lead to an underestimation of the impact of BI, and these factors need to be minimized as much as possible. The design of the SMART-ED study allows determination of the incremental contribution of the assessment process to the change in subsequent substance use, although it is not able to address or eliminate other sources of potential bias. The ability to disaggregate the effects attributable to assessment versus the BI is methodologically important and allows a clearer perspective of the mechanism of action in the observed change in alcohol or drug use.

From both a clinical and public health perspective, however, this methodological disaggregation of relative effects may be less important. The SBIRT approach has typically followed a stepped-care approach; some form of screening, often asking as few as one question [58], is used for case-finding purposes. In a number of general health-care settings, a positive response to such a screening item might be sufficient to lead directly to BI and/or advice to change behavior without conducting a more extended assessment. In other settings, an assessment (even brief) is conducted with patients who score positive for problem substance use to provide additional information about the nature and severity of use beyond the initial screen, after which a brief motivationally focused intervention is conducted, usually incorporating feedback based on information derived from the assessment. Assessment is considered to be an integral component of the intervention “bundle” in such an approach. Thus, if integrated into the intervention, and if found to contribute independently to the change process, assessments could be designed to maximize the therapeutic benefit they provide and add to the overall intervention effectiveness [9].

## Competing interests

The authors declare that they have no competing interests.

## Authors’ contributions

All authors participated in the conceptualization and design of the protocol. DMD developed the initial draft of the manuscript; all authors read, provided editorial input, and approved the final manuscript.

## References

[B1] US Institute of Medicine Broadening the Base of Treatment for Alcohol Problems 1990 National Academy Press, Washington, DC 25032399

[B2] Babor TF McRee BG PAK Grimaldi PL Ahmed K Bray J Screening, brief intervention, and referral to treatment (SBIRT): Toward a public health approach to the management of substance abuse Subst Abuse 2007 28 3 7 30 10.1300/J465v28n03_03 18077300

[B3] Bernstein J Bernstein E Tassiopoulos K Heeren T Levenson S Hingson R Brief motivational intervention at a clinic visit reduces cocaine and heroin use Drug Alcohol Depend 2005 77 1 49 59 10.1016/j.drugalcdep.2004.07.006 15607841

[B4] Babor TF Kadden RM Screening and interventions for alcohol and drug problems in medical settings: What works? J Trauma 2005 59 3 Suppl S80 S87 16355071 10.1097/01.ta.0000174664.88603.21

[B5] Madras BK Compton WM Avula D Stegbauer T Stein JB Clark HW Screening, brief interventions, referral to treatment (SBIRT) for illicit drug and alcohol use at multiple healthcare sites: comparison at intake and 6 months later Drug Alcohol Depend 2009 99 1–3 280 295 18929451 10.1016/j.drugalcdep.2008.08.003 PMC2760304

[B6] Dunn C Brief motivational interviewing interventions targeting substance abuse in the acute care medical setting Semi Clin Neuropsychiatr 2003 8 3 188 196 10.1016/S1084-3612(03)00025-X 12874739

[B7] Miller WM, Rollnick S Motivational Interviewing: Preparing People to Change Addictive Behavior 1991 Guilford Press, New York

[B8] Babor TF Higgins-Biddle JC Brief Intervention for Hazardous and Harmful Drinking: A Manual for Use in Primary Care 2001 World Health Organization, Geneva

[B9] Schrimsher GW Filtz K Assessment reactivity: Can assessment of alcohol use during research be an active treatment? Alcoholism Treat Q 2011 29 108 115 10.1080/07347324.2011.557983

[B10] Kypri K Langley JD Saunders JB Cashell-Smith ML Assessment may conceal therapeutic benefit: findings from a randomized controlled trial for hazardous drinking Addiction 2007 102 1 62 70 10.1111/j.1360-0443.2006.01632.x 17207124

[B11] McCambridge J Butor-Bhavsar K Witton J Elbourne D Can research assessments themselves cause bias in behaviour change trials? A systematic review of evidence from Solomon 4-group studies PLoS One 2011 6 10 e25223 10.1371/journal.pone.0025223 22039407 PMC3198466

[B12] McCambridge J Day M Randomized controlled trial of the effects of completing the Alcohol Use Disorders Identification Test questionnaire on self-reported hazardous drinking Addiction 2008 103 2 241 248 10.1111/j.1360-0443.2007.02080.x 18199302

[B13] McCambridge J Kypri K Can simply answering research questions change behaviour? Systematic review and meta analyses of brief alcohol intervention trials PLoS One 2011 6 10 e23748 10.1371/journal.pone.0023748 21998626 PMC3187747

[B14] Walters ST Vader AM Harris TR Jouriles EN Reactivity to alcohol assessment measures: an experimental test Addiction 2009 104 8 1305 1310 10.1111/j.1360-0443.2009.02632.x 19624323 PMC2724752

[B15] Bernstein JA Bernstein E Heeren TC Mechanisms of change in control group drinking in clinical trials of brief alcohol intervention: Implications for bias toward the null Drug Alcohol Rev 2010 29 5 498 507 10.1111/j.1465-3362.2010.00174.x 20887573

[B16] Clifford PR Maisto SA Subject reactivity effects and alcohol treatment outcome research J Stud Alcohol 2000 61 6 787 793 11188483 10.15288/jsa.2000.61.787

[B17] Clifford PR Maisto SA Davis CM Alcohol treatment research assessment exposure subject reactivity effects: part I. Alcohol use and related consequences J Stud Alcohol Drugs 2007 68 4 519 528 17568955 10.15288/jsad.2007.68.519

[B18] Clifford PR Maisto SA Franzke LH Longabaugh R Beattie MC Alcohol treatment research follow-up interviews and drinking behaviors J Stud Alcohol 2000 61 5 736 743 11022814 10.15288/jsa.2000.61.736

[B19] Epstein EE Drapkin ML Yusko DA Cook SM McCrady BS Jensen NK Is alcohol assessment therapeutic? Pretreatment change in drinking among alcohol-dependent women J Stud Alcohol 2005 66 3 369 378 16047526 10.15288/jsa.2005.66.369

[B20] Kaminer Y Burleson JA Burke R Can assessment reactivity predict treatment outcome among adolescents with alcohol and other substance use disorders? Subst Abuse 2008 29 2 63 69 10.1080/08897070802093262 19042325

[B21] Morton V Torgerson DJ Regression to the mean: treatment effect without the intervention J Eval Clin Pract 2005 11 1 59 65 10.1111/j.1365-2753.2004.00505.x 15660538

[B22] Moos RH Context and mechanisms of reactivity to assessment and treatment Addiction 2008 103 2 249 250 18199303 10.1111/j.1360-0443.2007.02123.x

[B23] Cherpitel CJ Korcha RA Moskalewicz J Swiatkiewicz G Ye Y Bond J Screening, brief intervention, and referral to treatment (SBIRT): 12-month outcomes of a randomized controlled clinical trial in a Polish emergency department Alcohol Clin Exp Res 2010 34 11 1228 1922 10.1111/j.1530-0277.2010.01281.x PMC2965307 20659072

[B24] Daeppen JB Gaume J Bady P Yersin B Calmes JM Givel JC Gmel G Brief alcohol intervention and alcohol assessment do not influence alcohol use in injured patients treated in the emergency department: a randomized controlled clinical trial Addiction 2007 102 8 1224 1233 10.1111/j.1360-0443.2007.01869.x 17565563

[B25] Bernstein E Edwards E Dorfman D Heeren T Bliss C Bernstein J Screening and brief intervention to reduce marijuana use among youth and young adults in a pediatric emergency department Acad Emerg Med 2009 16 11 1174 1185 10.1111/j.1553-2712.2009.00490.x 20053238 PMC2910362

[B26] Bogenschutz MP Donovan DM Adinoff B Crandall C Forcehimes AA Lindblad R Mandler RN Oden NL Perl HI Walker R Design of NIDA CTN Protocol 0047: screening, motivational assessment, referral, and treatment in emergency departments (SMART-ED) Am J Drug Alcohol Abuse 2011 37 5 417 425 10.3109/00952990.2011.596971 21854285 PMC3168577

[B27] Fals-Stewart W O’Farrell TJ Freitas TT McFarlin SK Rutigliano P The timeline followback reports of psychoactive substance use by drug-abusing patients: Psychometric properties J Consult Clin Psychol 2000 68 1 134 144 10710848 10.1037//0022-006x.68.1.134

[B28] Sobell LC Sobell MB Timeline Followback User’s Guide: A Calendar Method for Assessing Alcohol and Drug Use 1996 Addiction Research Foundation, Toronto

[B29] NIDA-Modified Alcohol, Smoking, and Substance Involvement Screening Test: NM-ASSISThttp://www.drugabuse.gov/nidamed/nmassist-screening-tobacco-alcohol-other-drug-use

[B30] McLellan AT Alterman AI Cacciola J Metzger D A new measure of substance abuse treatment: Initial studies of the Treatment Services Review J Nerv Ment Dis 1992 180 2 101 110 10.1097/00005053-199202000-00007 1737971

[B31] Diaz FJ Jané M Saltó E Pardell H Salleras L Pinet C de Leon J A brief measure of high nicotine dependence for busy clinicians and large epidemiological surveys Aust NZ J Psychiatry 2005 39 3 161 168 10.1080/j.1440-1614.2005.01538.x 15701065

[B32] Bush K Kivlahan DR McDonell MB Fihn SD Bradley KA The AUDIT alcohol consumption questions (AUDIT-C): an effective brief screening test for problem drinking Arch Intern Med 1998 158 16 1789 1795 10.1001/archinte.158.16.1789 9738608

[B33] Saunders JB Aasland OG Babor TF de-la-Fuente JR Grant M Development of the Alcohol Use Disorders Identification Test (AUDIT): WHO collaborative project on early detection of persons with harmful alcohol consumption: II Addiction 1993 88 6 791 804 10.1111/j.1360-0443.1993.tb02093.x 8329970

[B34] Skinner HA The drug abuse screening test Addict Behav 1982 7 4 363 371 10.1016/0306-4603(82)90005-3 7183189

[B35] Yudko E Lozhkina O Fouts A A comprehensive review of the psychometric properties of the Drug Abuse Screening Test J Substance Abuse Treat 2007 32 2 189 198 10.1016/j.jsat.2006.08.002 17306727

[B36] Gavin D Ross H Skinner H The diagnostic validity of the Drug Abuse Screening Test in the assessment of DSM-III drug disorders Br J Addict 1989 84 301 307 10.1111/j.1360-0443.1989.tb03463.x 2650770

[B37] Humeniuk R Ali R Babor TF Farrell M Formigoni ML Jittiwutikarn J de Lacerda RB Ling W Marsden J Monteiro M Validation of the alcohol, smoking and substance involvement screening test (ASSIST) Addiction 2008 103 6 1039 1047 10.1111/j.1360-0443.2007.02114.x 18373724

[B38] Caplan YH Goldberger BA Alternative specimens for workplace drug testing J Anal Toxicol 2001 25 396 399 11499896 10.1093/jat/25.5.396

[B39] Gallardo E Queiroz JA The role of alternative specimens in toxicological analysis Biomed Chromatogr 2008 22 795 821 10.1002/bmc.1009 18506679

[B40] Kintz P Villain M Cirimele V Hair analysis for drug detection Ther Drug Monit 2006 28 442 446 10.1097/01.ftd.0000211811.27558.b5 16778731

[B41] Dunn C Ostafin B Brief interventions for hospitalized trauma patients J Trauma 2005 59 3 Suppl S88 S93 16355072 10.1097/01.ta.0000174682.13138.a3

[B42] Dunn CW Donovan DM Gentilello LM Practical guidelines for performing alcohol interventions in trauma centers J Trauma 1997 42 299 304 10.1097/00005373-199702000-00021 9042886

[B43] Gentilello LM Donovan DM Dunn CW Rivara FP Alcohol interventions in trauma centers: current practice and future directions JAMA 1995 274 13 1043 1048 10.1001/jama.1995.03530130049027 7563455

[B44] Gentilello LM Rivara FP Donovan DM Jurkovich GJ Daranciang E Dunn CW Villaveces A Copass M Ries R Alcohol interventions in a trauma center as a means of reducing the risk of injury recurrence Ann Surg 1999 230 4 473 480 10.1097/00000658-199910000-00003 10522717 PMC1420896

[B45] Dunn C Deroo L Rivara FP The use of brief interventions adapted from motivational interviewing across behavioral domains: a systematic review Addiction 2001 96 12 1725 1742 10.1046/j.1360-0443.2001.961217253.x 11784466

[B46] Field C Hungerford DW Dunn C Brief motivational interventions: an Introduction J Trauma 2005 59 3 Suppl S21 S26 16355056 10.1097/01.ta.0000179899.37332.8a

[B47] Krupski A Sears JM Joesch JM Estee S He L Dunn C Huber A Roy-Byrne P Ries R Impact of brief interventions and brief treatment on admissions to chemical dependency treatment Drug Alcohol Depend 2010 110 1–2 126 136 20347234 10.1016/j.drugalcdep.2010.02.018

[B48] Estee S Wickizer T He L Shah MF Mancuso D Evaluation of the Washington State screening, brief intervention, and referral to treatment project: cost outcomes for Medicaid patients screened in hospital emergency departments Medical Care 2010 48 1 18 24 10.1097/MLR.0b013e3181bd498f 19927016

[B49] Dunn CW Estee S Huber A Ries R Screening, brief intervention, and referral to treatment for substance abuse: Bringing substance abuse counseling to acute medical care. A training manual for staff in acute medicine settings 2010 Washington State Department of Social and Health Services, Olympia, WA

[B50] Sise MJ Sise CB Kelley DM Simmons CW Kelso DJ Implementing screening, brief intervention, and referral for alcohol and drug use: the trauma service perspective J Trauma 2005 59 3 Suppl S112 S118 16355046 10.1097/01.ta.0000176045.95492.01

[B51] Mello MJ Longabaugh R Baird J Nirenberg T Woolard R DIAL: a telephone brief intervention for high-risk alcohol use with injured emergency department patients Ann Emerg Med 2008 51 6 755 764 10.1016/j.annemergmed.2007.11.034 18436341

[B52] Miller WR Zweben A Diclemente CC Rychtarik RG Motivational enhancement therapy manual: A clinical research guide for therapists treating individuals with alcohol abuse and dependence 1992 National Institute on Alcohol Abuse and Alcoholism, Rockville, MD

[B53] Longabaugh R Woolard RE Nirenberg TD Minugh AP Becker B Clifford PR Carty K Sparadeo F Gogineni A Evaluating the effects of a brief motivational intervention for injured drinkers in the emergency department J Stud Alcohol 2001 62 6 806 816 11838918 10.15288/jsa.2001.62.806

[B54] Bischof G Grothues JM Reinhardt S Meyer C John U Rumpf H-J Evaluation of a telephone-based stepped care intervention for alcohol-related disorders: A randomized controlled trial Drug Alcohol Depend 2008 93 3 244 251 10.1016/j.drugalcdep.2007.10.003 18054443

[B55] Curry SJ Ludman EJ Grothaus LC Donovan D Kim E A randomized trial of a brief primary-care-based intervention for reducing at-risk drinking practices Health Psychol 2003 22 2 156 165 12683736

[B56] Estee S He L Use of Alcohol and Other Drugs Declined among Emergency Department Patients Who Received Brief Interventions for Substance Use Disorders through WASBIRT 2007 Washington State Department of Social and Health Services, Olympia, WA

[B57] Smith PC Schmidt SM Allensworth-Davies D Saitz R Primary care validation of a single-question alcohol screening test J Gen Intern Med 2009 24 7 783 788 10.1007/s11606-009-0928-6 19247718 PMC2695521

[B58] Smith PC Schmidt SM Allensworth-Davies D Saitz R A single-question screening test for drug use in primary care Arch Intern Med 2010 170 13 1155 1160 10.1001/archinternmed.2010.140 20625025 PMC2911954

[B59] de Leon J Diaz FJ Becona E Gurpegui M Jurado D Gonzalez-Pinto A Exploring brief measures of nicotine dependence for epidemiological surveys Addict Behav 2003 28 8 1481 1486 10.1016/S0306-4603(02)00264-2 14512071

